# Supplementary material for: Alkaline Dilution Alters Sperm Motility in Dairy Goat by Affecting sAC/cAMP/PKA Pathway Activity
Source: Int J Mol Sci. 2023 Jan 16;24(2):1771. doi: 10.3390/ijms24021771 (PMC9863640; doi:10.3390/ijms24021771)

Supplementary_Material 2

1.

For cell-free measurements of adenylate cyclase activity, washed cauda epididymal spermatozoa were resuspended in lysis buffer and sonicated three times for 30 s each (Branson Sonifier 450) at a power setting of 6 with alternating 30-s periods of ice cooling. Assay conditions were as described in Jaiswal and Conti [24]. Briefly, sonicates were incubated in 100 µl of reaction buffer containing 40 mM Tris–HCl, pH 7.5, 5 mM MgCl2, 0.2 mM cAMP, 10 mM phosphoenolpyruvate, 3 units of pyruvate kinase, 10 µM GTP, 1 mM ATP, and 2 µCi of α-[^{32}P]-ATP for 20 min at 37°C. Reaction was terminated with the addition of 20 µl of 2.2 N HCl containing [^{3}H]-cAMP (0.01 µCi) followed by boiling for 4 min and then cooling in an ice water bath. Labeled cAMP was added to estimate and corrected for recovery of the cyclic nucleotide during column chromatography. The cAMP was then separated from ATP on alumina columns eluted with 5 ml of 0.1 M ammonium acetate, pH 6.5.

2.

For cAMP evaluation, the cAMP direct immunoassay fluorometric kit (abcam, Cambridge, United Kingdom; ab138880) was used, following the manufacturer's specifications. In brief, Sperm incubated under different conditions were centrifuged at 3000× g for 5 min at room temperature (RT); then, the supernatant was discarded, and the pellet was resuspended by adding 100 µL of Cell Lysis Buffer in each well of the plate and incubated at RT for 10 min. Samples were centrifuged for 5 min at top speed, and the supernatant was collected and transferred to a new tube that was kept on ice. A volume of 75 µL of standard and sample was added to the wells of the anti-cAMP coated 96-well plate and incubated at RT for 5–10 min. After this time, 25 µL/well of 1X HRP-cAMP conjugate was added to each standard and sample well. Then, the plate was incubated at RT for 2 h on a plate shaker and washed 4 times with 200 µL Wash Solution. Finally, 100 µL AbRed Working Solution was added into each standard and sample well, incubating the plate at RT for 1 h protected from light. Fluorescence change was measured in a microplate reader set to top read mode at Ex/Em = 540/590

nm (cutoff 570 nm).

3.

For PKA evaluation, we used the PKA Kinase Activity Assay Kit (abcam, Cambridge, United Kingdom; ab139435) according to the manufacturer's instructions. In brief, Sperm incubated under different conditions were centrifuged at 3000× g for 5 min at RT, and the pellet was resuspended in 100 µL of RIPA. After sonication of the samples (10 s twice) and 5 s vortexing, the samples were kept on ice for 30 min. Then, the lysate was centrifuged 13,000× g for 15 min at 4 ◦C. After recovery of a clear supernatant, the samples were kept on ice. Wells were previously prepared with 50 µL Kinase Assay Dilution Buffer at RT for 10 min. Then, 30 µL Kinase dilution buffer was added to the blank wells, samples (30 µL) and controls (30 µL) to the appropriate wells. An amount of 10 µL of diluted ATP to each well (except blank) initiates the reaction, after incubation at 30 ◦C for 90 min. Then, 40 µL of phospho-specific antibody was added to each well, except blank, and the plate was incubated at RT for 60 min. All wells were washed 4 times with 100 µL 1X Wash Buffer. Afterward, 40 µL of diluted anti-rabbit IgG-HRP conjugated was added to each well except blank, and the plate was incubated at RT for 30 min and washed 4 times with wash buffer. Thereafter, 60 µL TMB solution was added and incubated for 45 min at RT. Finally, 20 µL stop solution was added to each well to stop the reaction, and the optical density (OD) at 450 nm was measured.

4. Assessment of capacitation status of enriched sperm

The sperm capacitation status was assessed using the CTC fluorescence assay method, as described previously [25]. CTC is a fluorescent antibiotic whose distribution in the spermatozoa changes during the transition from non-capacitated to capacitated and then to acrosome-reacted state, thereby allowing to differentiation various steps of the sperm capacitation process [26]. Briefly, spermatozoa (1 × 106 cells/ml) were suspended in TALP and centrifuged at 500×*g* for 5 min. The spermatozoa pellet obtained was washed thrice with TALP and then the pellet was suspended in 0.5 ml of TALP to serve as the stock of spermatozoa solution. To spermatozoa suspension, 250 µL of CTC stock (1 mg/ml) solution was added and incubated in dark at 37 °C for 30 min.

After incubation, 5 μL of CTC-labeled spermatozoa suspension was mixed with 5 μL antifade medium (1.5% w/v DABCO in 90% v/v glycerol) and placed on a glass slide for examination using Nikon Eclipse TE 2000-S microscope with phase contrast and epifluorescence optics under blue-violet illumination (excitation at 400–440 nm and emission at 470 nm by using 40× objectives). A total of 400 spermatozoa per slide were observed and different patterns CTC reactive spermatozoa were evaluated.

5 Measurement of phosphorylation of PKA kinase substrates

The PKA activity kit is designed to quantitatively measure PKA activity in a variety of samples. A recombinant PKA standard is provided to generate a standard curve for the assay and all samples should be read off the standard curve. The kit utilizes an immobilized PKA substrate bound to a microtiter plate. Samples containing PKA will, in the presence of the supplied ATP, phosphorylate the immobilized PKA substrate. After a 90-minute incubation followed by a wash, a rabbit antibody specific for the phospho-PKA substrate binds to the modified immobilized substrate. An antibody specific for rabbit IgG labeled with peroxidase is also added to the plate to bind to the rabbit anti-phospho-PKA substrate. After a short incubation and wash, substrate is added and the intensity of the color developed is directly proportional to the amount of PKA in the samples and standards.

According to the instructions of the Protein Kinase A (PKA) Colorimetric Activity Kit (EIAPKA, ThermoFisher Scientific), A standard curve of PKA kinase activity versus Optical Density (450nm) was established as shown below.

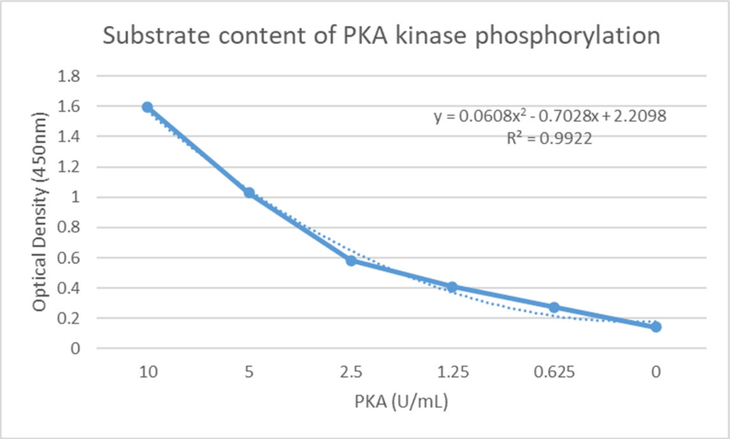

6. Determination of substrate content of tyrosine phosphorylation

For pY evaluation, we used the Anti-Phosphotyrosine Polyclonal Antibody Elisa Kit (abs120596, Absin) according to the manufacturer's instructions. In brief, Sperm incubated under different conditions were centrifuged at 3000× g for 5 min at RT, and the pellet was resuspended in 100 µL of RIPA. After sonication of the samples (10 s twice) and 5 s vortexing, the samples were kept on ice for 30 min. Then, the lysate was centrifuged 13,000× g for 15 min at 4 ◦C. After recovery of a clear supernatant, the samples were kept on ice. Wells were previously prepared with 50 µL Kinase Assay Dilution Buffer at RT for 10 min. Then, 30 µL Kinase dilution buffer was added to the blank wells, samples (30 µL) and controls (30 µL) to the appropriate wells. Then, 40 µL of phospho-specific antibody was added to each well, except blank, and the plate was incubated at RT for 60 min. All wells were washed 4 times with 100 µL 1X Wash Buffer. Afterward, 40 µL of diluted anti-rabbit IgG-HRP conjugated was added to each well except blank, and the plate was incubated at RT for 30 min and washed 4 times with wash buffer. Thereafter, 60 µL TMB solution was added and incubated for 45 min at RT. Finally, 20 µL stop solution was added to each well to stop the reaction, and the optical density (OD) at 450 nm was measured.

According to the instructions of the Anti-Phosphotyrosine Polyclonal Antibody Elisa Kit (abs120596, Absin), The standard curve of tyrosine phosphorylated substrate content versus optical density at 450nm is shown below.

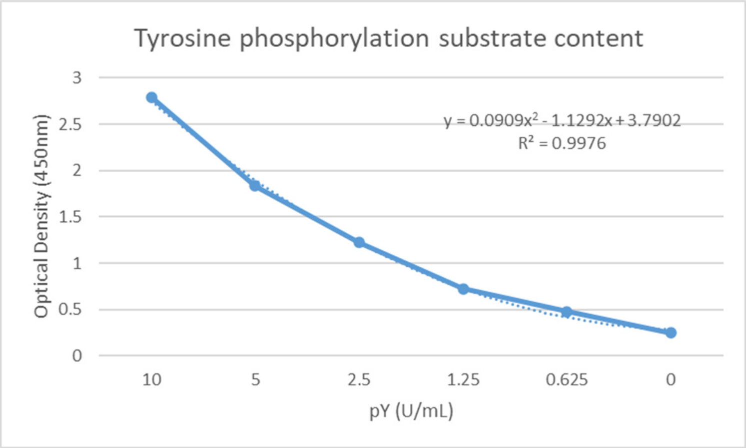

Supplement: Supplementary file 1 [file ijms-24-01771-s001.zip › Supplementary_Material 2.pdf]
